# Exploration of lymph node recurrence patterns and delineation guidelines of radiation field in middle thoracic oesophageal carcinomas after radical surgery: a real-world study

**DOI:** 10.1186/s12885-024-12297-4

**Published:** 2024-05-16

**Authors:** Rongxu Du, Songqing Fan, Dan Yang, Xiaobin Wang, Xia Hou, Cheng Zeng, Dan Guo, Rongrong Tian, Leilei Jiang, Xin Dong, Rong Yu, Huiming Yu, Shuchai Zhu, Jie Li, Anhui Shi

**Affiliations:** 1 Key laboratory of Carcinogenesis and Translational Research (Ministry of Education/Beijing), Department of Radiation Oncology, Peking University Cancer Hospital & Institute, Beijing, 100142 China; 2 Oncology Division I, China Pingmei Shenma Medical Group General Hospital, Kuanggongzhong Rd.1, Xinhua District, Pingdingshan Henan, 450052 China; 3 Department of Radiation Oncology, Hebei Cancer Hospital, The Fourth Hospital of Hebei Medical University, JianKang Rd.12, Shijiazhuang Hebei, 050011 China; 4 Department of Radiation Oncology, Shanxi Provincial Cancer Hospital, No.3 Workers New Village, Xinghualing District, Taiyuan, Shanxi 030013 China; 5 Department of Radiation Oncology, Central Theater General Hospital, Wuluo Rd. 627, Wuchang District, Wuhan Hubei, 430061 China

**Keywords:** Middle thoracic oesophageal carcinoma, Postoperative lymph node recurrence, Radiation field, Real-world study

## Abstract

**Background:**

Oesophageal squamous cell carcinoma is one of the most commonly diagnosed carcinomas in China, and postoperative radiotherapy plays an important role in improving the prognosis of patients. Carcinomas in different locations of the oesophagus could have different patterns of lymph node metastasis after surgery.

**Methods:**

In this multicentric retrospective study, we enrolled patients with middle thoracic oesophageal squamous cell carcinomas from 3 cancer centres, and none of the patients underwent radiotherapy before or after surgery. We analysed the lymph node recurrence rates in different stations to explore the postoperative lymphatic recurrence pattern.

**Results:**

From January 1st, 2014, to December 31st, 2019, 132 patients met the criteria, and were included in this study. The lymphatic recurrence rate was 62.1%. Pathological stage (*P* = 0.032) and lymphadenectomy method (*P* = 0.006) were significant predictive factors of lymph node recurrence. The recurrence rates in the supraclavicular, upper and lower paratracheal stations of lymph nodes were 32.6%, 28.8% and 16.7%, respectively, showing a high incidence. The recurrence rate of the subcarinal node station was 9.8%, while 8.3% (upper, middle and lower) thoracic para-oesophageal nodes had recurrences.

**Conclusions:**

We recommend including the supraclavicular, upper and lower paratracheal stations of lymph nodes in the postoperative radiation field in middle thoracic oesophageal carcinomas. Subcarinal station is also potentially high-risk, while whether to include thoracic para-oesophageal or abdominal nodes needs careful consideration.

## Background

Oesophageal carcinoma is one of the most commonly diagnosed carcinomas in the world. In 2020, there were an estimated 604,000 cases, and there were more than 544,000 deaths worldwide, making it the 7th most commonly diagnosed cancer and the 6th main cause of cancer-related deaths worldwide [1, 2]. The percentage of diagnosed cases and deaths from China could be as high as 50% of all oesophageal carcinoma cases in the world per year [3]. While the overwhelming majority of the cases in our country and even in Asia are squamous cell carcinomas, adenocarcinomas make up most of the cases in the United States and western Europe [4, 5].

Although comprehensive treatment has been recommended for oesophageal carcinomas, compared to the high popularity rate of preoperative chemoradiation, which has exceeded 40% in the United States during the last decade, the application of neoadjuvant treatment in China was not that widespread [6]. According to a study from the Oesophageal Cancer Committee of the China Anti-Cancer Association, the ratios of neoadjuvant radiotherapy, neoadjuvant chemotherapy, postoperative radiotherapy and postoperative chemotherapy were reported to be approximately 2.0%, 2.0%, 7.0% and 26.0%, respectively, in 2012 [7]. Adjuvant radiotherapy has been shown to be beneficial for decreasing recurrence and prolonging overall survival, especially in stage II-III patients and pN + patients [8–13]. Considering the fact that oesophageal carcinoma patients in our country have been more likely to undergo surgery as the first step of treatment, adjuvant radiotherapy could play a more important role in the treatment of the Chinese oesophageal carcinoma population. According to the Chinese guidelines on the radiotherapy of oesophageal carcinomas [14], for middle thoracic oesophageal carcinomas, postoperative radiation field was recommended to include supraclavicular, paratracheal, subcarinal and upper thoracic para-oesophageal stations of lymph nodes.

Due to the heterogeneity of clinical characteristics and treatment strategies in diverse regions in our country, it is necessary to describe the real-world recurrence patterns of the oesophageal carcinomas in China.

## Methods

This retrospective study included patients diagnosed with middle thoracic oesophageal cancers who underwent curative surgery at the Department of Thoracic Surgical Oncology of 3 clinical centres, including Beijing Cancer Hospital, Hebei Province Cancer Hospital and Shanxi Province Cancer Hospital, from January 1st, 2014, to December 31st, 2019. According to the 8th edition of the American Joint Committee on Cancer (AJCC) criteria, the location of the primary tumour is defined by the centre of the tumour, while the middle thoracic segment is defined as the part of the oesophagus from the azygos vein to the inferior pulmonary vein (25 cm to 30 cm away from the incisors, measured by endoscopy) [15]. The lymph node groups included in our study were also defined by the 8th edition of the AJCC criteria [15].

### Patients

The inclusion criteria for enrollment were as follows: (1) patients aged 18 to 80 years; (2) patients with tumours that were confirmed by the postoperative pathological results to be clearly diagnosed as squamous cell oesophageal carcinomas; (3) patients with tumours confirmed to be located on the middle segment of the thoracic oesophagus by endoscopy; (4) patients confirmed to have R0 resection by postoperative pathology; (5) patients with sufficient imaging materials covering all the treatment history and follow-up; and (6) lymphatic recurrence confirmed by PET-CT or continuous enhanced computed tomography (CT) scan. All the patients accepted upper digestive tract radiography, CT and endoscopy examination before surgery to define the clinical stage and make treatment decisions. For patients with high risk of distant metastasis or invading surrounding organs, PET-CT and ultrasound bronchoscopy were also recommended.

The exclusion criteria included the followings: (1) patients histologically diagnosed with nonsquamous cell histological types; (2) patients with cervical oesophageal carcinomas or upper/lower thoracic oesophageal carcinomas; (3) patients who underwent perioperative radiation; (4) patients with more than one primary tumour; and (5) patients lacking important clinical information, e.g., pathological results, surgical records or imaging results.

We acquired the clinical data in a retrospective manner, and our study has been examined and approved by the Ethics Committee of Beijing Cancer Hospital.

### Assessment

^18^F-fluorodeoxyglucose positron emission tomography (FDG-PET) has been broadly used to detect lymph node metastasis before treatment and lymph node recurrence later [16]. Our research also used FDG-PET to evaluate and confirm lymph node recurrence, and lymph nodes with a clear, high uptake of FDG in PET-CT (SUV ≥ 2.5) were determined to be recurrent. For patients who failed to undergo PET-CT due to personal reasons, continuous enhanced CT was regarded as an alternative imaging method. The criteria for CT evaluation were as follows: lymph node with a short axis greater than 1 cm, tracheoesophageal groove lymph node with a short axis greater than 0.5 cm, or suspicious nodes that gradually enlarged during the observation period, all of which were shown by the enhanced CT images. The patients who failed to meet the criteria above but were still highly suspected to have recurrence were confirmed by biopsy and pathological diagnosis or were evaluated via a multidisciplinary team (MDT).

The first time when lymph node recurrence was recorded by PET-CT or continuous enhanced CT scan after completion of radical treatments (including surgery and perioperative chemotherapy) or the end of the follow-up was set as the endpoint.

### Statistical analysis

We used SPSS 24.0 (SPSS Inc., Chicago, IL, USA) to organize the data and perform statistical analysis. We set a cut-off value of 10% to distinguish the lymph node stations with high risk of recurrence from the others. The chi-square test was used to determine the factors related to lymph node recurrence, and binary regression logistic analysis was conducted for risk factors related to lymphatic recurrence. All variables with significant results as determined by the univariate analysis (*P* < 0.05) were included in the multivariate model.

## Results

From January 1st, 2014, to December 31st, 2019, 132 patients with middle thoracic oesophageal carcinomas were included in this study.

The patients were aged from 43 to 80 years (median age of 62), and male patients still constituted a major proportion of our study population, accounting for 77.3% of the patients. Twenty-eight patients underwent adjuvant chemotherapy, and 10 patients underwent neoadjuvant chemotherapy. Among the cohort, 44 patients (33.3%) had stage III-IV diseases indicated by postoperative pathology. It is worth noting that 59.8% of the included patients underwent oesophagectomy with three-field (cervical-thoracic-abdominal) lymphadenectomy, 21.2% underwent oesophagectomy with two-field (thoracic-abdominal) lymphadenectomy, and the remaining 19% underwent transhiatal surgery or unspecified surgical methods. The basic characteristics of the patients were listed in Table 1.

**Table 1:** Baseline characteristics of the enrolled patients

Characteristics	Patients (*N* = 132)		
	Number	Constituent ratio (%)	*P* value in chi-square analysis of recurrence
**Age**	43y-80 y (62 y)	-	-
**Sex**			0.259
Male	102	77.3%	
Female	30	22.7%	
**Pathological type**			-
Squamous cell carcinomas	132	100%	
**Invasion depth**			0.561
(y)pT0-T2	65	49.2%	
(y)pT3-T4	67	50.8%	
**Stage (AJCC 8th)**			0.007
Stage 0	3	2.3%	
Stage I	22	16.7%	
Stage II	63	47.7%	
Stage III	38	28.8%	
Stage IV	6	4.5%	
**Grade of differentiation**			0.510
Highly differentiated	10	7.6%	
Moderately differentiated	68	51.5%	
Poorly differentiated	54	40.9%	
**Location**			-
Middle thoracic	132	100%	
**Chemotherapy**			0.428
Neoadjuvant+/adjuvant chemotherapy	37	28.0%	
Without chemotherapy	95	72.0%	
**Lymphadenectomy method**			0.009
Two-field	28	21.2%	
Three-field	79	59.8%	
Other methods or undefined methods	25	19.0%	
**Anastomosis condition**			0.051
Anastomotic recurrence	11	8.3%	
Without defined anastomotic recurrence	121	91.7%	
**Concurrent distant metastasis**			0.000
Yes	24	18.2%	
No	108	81.8%	
**Recurrence**			-
LN recurrence	82	62.1%	
No LN recurrence	50	37.9%	
**Total**	132	100%	-

In summary, lymph node recurrences occurred in 82 patients. The lymphatic recurrence rate was 62.1%. We evaluated the factors related to recurrence, as shown in Tables 1 and 2. In the multivariate analysis, pathological stage (*P* = 0.032, HR = 2.847, 95% CI 1.097–7.391) and surgical method were indicated to be of prognostic value in predicting recurrence. Notably, compared to three-field lymphadenectomy, two-field lymphadenectomy was related to a higher risk of recurrence (*P* = 0.006, HR = 6.264, 95% CI 1.844–21.282).

**Table 2:** Multivariate analysis in the prognosis factors of lymph node recurrence

Prognosis factors	*P* value	HR	95% CI
Pathological stage III-IV vs. stage 0-II	0.032	2.847	1.097–7.391
Two-field lymphadenectomy vs. three-field lymphadenectomy	0.006	6.264	1.844–21.282

**Fig. 1:**
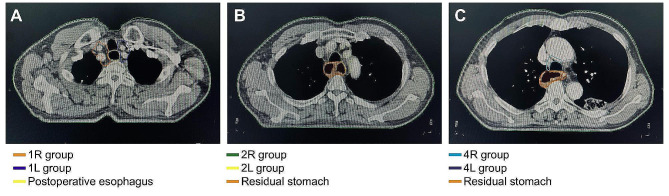
An example of delineation of the radiation field of patients with middle thoracic oesophageal carcinomas

More importantly, our results illustrated the distribution of recurrent lymph nodes after surgery in the population with middle thoracic oesophageal carcinomas (Table 3). The recurrence rates in the 1st (supraclavicular) and 2nd (upper paratracheal) stations of lymph nodes were 32.6% and 28.8%, respectively, showing a high incidence. The 4th (lower paratracheal) station also had a high recurrence rate of 16.7%, and although the recurrence rates of the 7th (subcarinal) station of nodes (9.8%) and the 8th (thoracic para-oesophageal) station of nodes (8.3%) didn’t exceed the cut-off value set in our study, the ratios of recurrent nodes were relatively high on the whole. Although the abdominal lymph nodes (the 16th -20th stations) showed a recurrence rate of 9.0% as a whole, the rate of each station was generally low, as the 17th station showed a recurrence rate of 3.8%, which was the highest in the abdominal node stations. Based on the cut-off value of 10%, the 1st, 2nd and 4th stations could be at high risk of recurrence after radical surgery, and we presented an example of the delineation of the radiation field in Fig. 1.

**Table 3:** Distribution and rate of lymph node recurrence in middle thoracic oesophageal carcinomas

Lymph node stations	Number of patients with lymphatic recurrence		Percentage
1 (supraclavicular)	43	32.6%	
2 (upper paratracheal)	38	28.8%	
4 (lower paratracheal)	22	16.7%	
7 (subcarinal)	13	9.8%	
8U (upper thoracic para-oesophageal)	1	0.8%	
8M (middle thoracic para-oesophageal)	6	4.5%	
8Lo (lower thoracic para-oesophageal)	4	3.0%	
9 (pulmonary ligament)	0	0.0%	
16 (paracardial)	3	2.3%	
17 (along the left gastric artery)	5	3.8%	
18 (along the common hepatic artery)	2	1.5%	
19 (along the splenic artery)	0	0.0%	
20 (celiac)	3	2.3%	
Upper cervical	2	1.5%	

Due to the high incidence of recurrence in the 1st lymph node station, we additionally explored the relationship between possible high-risk variables and the occurrence of lymphatic recurrence in the 1st station, as shown in Table 4. We found that there was a significant difference between the patients with stage 0-II diseases and those with stage III-IV diseases for lymphatic recurrence in the 1st station (*P* = 0.038). There was no significant difference existing in other subgroups.

**Table 4:** Analysis of high-risk factors for recurrence in the 1st station

Variables	Patients with lymph node recurrences (*N* = 82)		
	Patients with recurrences in the 1st station	Patients without recurrences in the 1st station	*P* value
**Invasion depth**			0.666
(y)pT0-T2	23 (28.0%)	19 (23.2%)	
(y)pT3-T4	20 (24.4%)	20 (24.4%)	
**Pathological stage**			0.038
0-II	20 (24.4%)	27 (32.9%)	
III-IV	23 (28.0%)	12 (14.7%)	
**Grade of differentiation**			0.631
Highly differentiated	2 (2.4%)	3 (3.7%)	
Moderately differentiated	22 (26.8%)	23 (28.0%)	
Poorly differentiated	19 (23.2%)	13 (15.9%)	
**Anastomosis condition**			0.741
Anastomotic recurrence	6 (7.3%)	4 (4.9%)	
Without defined anastomotic recurrence	37 (45.1%)	35 (42.7%)	
**Concurrent distant metastasis**			0.097
Yes	16 (19.5%)	8 (9.8%)	
No	27 (32.9%)	31 (37.8%)	
**Condition of chemotherapy**			0.314
Neoadjuvant+/adjuvant chemotherapy	13 (15.9%)	8 (9.8%)	
Without chemotherapy	30 (36.5%)	31 (37.8%)	
**Lymphadenectomy method**			0.417
Two-field	11 (13.4%)	13 (15.9%)	
Three-field	25 (30.5%)	17 (20.7%)	
Other methods	7 (8.5%)	9 (11.0%)	

## Discussion

After curative oesophagectomy and lymphadenectomy, patients could still have a recurrence rate of approximately 27.1-52.6% [17], and lymph node recurrence has been a major form of recurrence, the rate of which can reach 42.9-57.9% [18]. Li et al. [19] compared survival and local failure in patient groups with surgery alone and surgery combined with different postoperative therapies. The 5-year overall survival (OS), disease-free survival (DFS) and locoregional recurrence were significantly worse in the surgery alone group (15.2%, 13.1% and 71.6%, all *P* < 0.05) than in the postoperative chemotherapy group (28.0%, 20.8% and 66.5%), postoperative radiotherapy group (27.4%, 24.4% and 46.9%), and postoperative chemoradiotherapy group (42.8%, 35.5% and 43.0%). Additionally, postoperative radiotherapy, combined with or without chemotherapy, decreased local recurrence (*P* < 0.01). Similarly, the study results from Ni et al. [20] suggested that postoperative radiotherapy increased the 5-year OS from 31.3 to 45.0% and the 5-year DFS from 24.2 to 39.8% in pN + oesophageal carcinoma patients. Li et al. [21] reported an even higher increase of approximately 20% in OS. And according to most studies, the improvement in OS could also be observed in patients with sequential or concurrent chemoradiation [9]. According to a Chinese retrospective study [18] involving 239 patients who underwent three-field lymph node dissection, patients had the highest recurrence rate in the mediastinal region, especially in the superior mediastinal region (67.72% in patients after surgery alone), followed by the cervical region. Less than 1/3 of the population in this study underwent postoperative radiotherapy, which reduced the recurrence rate of the superior mediastinal lymph nodes by 20% (*P* = 0.006).

Based on our previous real-world study [22], the recurrence patterns could be slightly different in practice, and the number of recurrent lymph nodes could influence the prognosis of patients. Comprehensive treatment, including surgery and postoperative radiotherapy, not only influenced the prognosis of patients but also the patterns of lymphatic recurrence. As a result, we paid more attention to lymph node recurrence patterns in patients without perioperative radiotherapy, which could present realistic patterns without influence from radiotherapy and give us more hints about postoperative radiation field. In a retrospective study [23] including 338 middle thoracic oesophageal carcinoma patients, the major site of lymphatic recurrence was the mediastinal region, followed by multiple sites, the supraclavicular region, the anastomotic site and the abdominal region. And a large-sample study indicated that the lymph node recurrences of middle thoracic cancers occurred most commonly in middle mediastinal [24]. As far as we know, the hospitals we chose were all large centres located in central China, where was representative of high incidence of oesophageal carcinomas in China [25], hence, the results of this study could represent the classical characteristics of oesophageal cancer in our country. And this study, as a sequential study of real-world research on lower thoracic oesophageal carcinomas [22], depicted the recurrence patterns of middle thoracic carcinomas and the high-risk prognostic factors of lymphatic metastasis. The recurrence patterns of carcinomas in the two different locations suggested that there were detailed differences in the frequency of recurrence in para-oesophageal and celiac lymph nodes, but both studies showed a high recurrence rate in the supraclavicular station.

In sequence, the recurrence rates of supraclavicular, upper paratracheal and lower paratracheal lymph nodes were 32.6%, 28.8% and 16.7%, which were above the cut-off value of 10% that we set, indicating a higher necessity of these stations to be included in the radiation field than the others. Admittedly, our results had discrepancies with classic studies and the clinical recommendations [26], which suggested that the 2nd, 4th, 7th, most of the 8th stations and part of the abdominal nodes should be included, while domestic guidelines [14] recommended including 1st, 2nd, 4th, 7th and 8U stations. Delineation of the radiation field for preventing recurrence has often been performed based on the findings during the operation, however, whether lymph node recurrence occurs in a consistent way before and after lymph node dissection remained questionable, and our study might propose other possibilities. First, we found that stage III-IV patients were more likely to have lymph node recurrences in the supraclavicular region, indicating that the supraclavicular station should be taken seriously in patients with advanced stages. Second, the low recurrence rates in the 8U nodes and 16th -20th nodes in our study suggested possible benefits from high-quality lymph node dissection. 81% of the patients in our study underwent sufficient dissection of the thoracic and abdominal lymph nodes, with or without cervical nodes. But due to the complex structure of the cervical region, which is abundant with vital organs, nerves and vessels, the concern regarding the damage to the function of structures and the difficulty in radical resection may result in tumour residues in the cervical region. Comparatively speaking, a thorough clearance of positive nodes in thoracic and abdominal regions is easier to achieve. If the lymphoid tissue is sufficiently resected during the operation, there might be only a slight chance of lymph node recurrence. Compared with the results from previous studies, our findings also suggested a high risk of recurrence in the 7th station. However, whether the 8U and abdominal regions should be contained in the adjuvant radiation field needed more evidence.

In addition, Law et al. [27] and Katayama et al. [28] reported an overall cervical lymphatic recurrence rate of 11-12%. The possibility of cervical lymphatic recurrence is part of the reason why three-field surgery method is preferred. Previous studies supported the clearance of nodes in the cervical region based on the considerable recurrence rate of the cervical region in middle thoracic oesophageal cancer, and this recurrence rate was reported to be 16-45.2% [29]. The 3-field surgical method was suggested to be more likely to result in postoperative complications due to the clearance of the cervical lymph nodes [30], which also indicated that there was difficulty in the thorough dissection of lymph nodes. However, Yamashita et al. [31] compared the clinical characteristics and prognosis of patients after 2-field and 3-field lymphadenectomy, and their results indicated that the patients who underwent 3-field surgeries always had more advanced stages, but number of dissected mediastinal and abdominal lymph nodes, did not differ significantly between the two groups. B. Li et al. reported that oesophagectomy with three-field lymphadenectomy increased the total number of lymph nodes dissected and resulted in stage migration owing to a rate of 21.5% in cervical lymph node metastasis [32]. The survival benefits of 3-field lymphadenectomy over 2-field lymphadenectomy have also been demonstrated in previous studies, especially in the middle thoracic cancer patients [33, 34]. Nearly 60% of the patients in our study underwent 3-field lymph node dissection, indicating a widespread application of this surgical method in middle thoracic oesophageal carcinoma, and the multivariate analysis demonstrated the advantage of the 3-field method over the 2-field method in reducing the risk of lymphatic recurrence.

Previous studies pointed out that several factors, such as grade of differentiation, depth of invasion, pathological stage, number of positive nodes and marginal status, were independent predictive factors of early recurrence and death after oesophageal carcinoma resection [35, 36]. The incidence of lymph node metastasis increases in more advanced stages, especially when the tumour infiltrates the submucosa [37], but is still high in patients with T1-T2 stage diseases. Our study also demonstrated the effect of the pathological stage on lymph node metastasis, which could be different in tumours in different locations of oesophagus.

Despite achieving applicable results from this research, there were some limitations of our study. Since we acquired most of the data retrospectively, some information was missing, and it was difficult to obtain sufficient materials from the patients’ clinical history, that was why we chose to analyze imaging data as a more reliable index. Besides, the sample size in our study was limited, our conclusions needed to be verified with a larger population or a prospective study.

## Conclusions

In conclusion, we recommend including the supraclavicular, upper and lower paratracheal stations of lymph nodes in the radiation field to prevent postoperative lymphatic recurrence in middle thoracic oesophageal carcinomas. Subcarinal station is also potentially high-risk, while whether to include thoracic para-oesophageal or abdominal nodes needs more careful consideration. After high-quality lymph node dissection, advanced stage suggests a higher possibility of recurrence in the supraclavicular station.

## Data Availability

All data generated or analyzed during this study used to support the findings of this study are included within the article.

## Abbreviations

AJCC: American Joint Committee on Cancer
MDT: Multiple-disciplinary team
CT: Computed tomography
FDG-PET: ^18^F-fluorodeoxyglucose positron emission tomography
OS: Overall survival
DFS: Disease-free survival
